# Analysis of Genetic Characterization and Clonality of *Legionella pneumophila* Isolated from Cooling Towers in Japan

**DOI:** 10.3390/ijerph16091664

**Published:** 2019-05-13

**Authors:** Noriko Nakanishi, Ryohei Nomoto, Shinobu Tanaka, Kentaro Arikawa, Tomotada Iwamoto

**Affiliations:** Department of Infectious Diseases, Kobe Institute of Health, 4-6-5 Minatojima-nakamichi, Chuo-ku, Kobe 650-0046, Japan; shinobu_tanaka@office.city.kobe.lg.jp (S.T.); kentaro_arikawa@office.city.kobe.lg.jp (K.A.); tomotada_iwamoto@office.city.kobe.lg.jp (T.I.)

**Keywords:** *Legionella pneumophila*, cooling tower, SBT, WGS, single nucleotide polymorphism (SNP)

## Abstract

We investigated the genetic characteristics of 161 *Legionella pneumophila* strains isolated over a period of 10 years from cooling towers in Japan. Minimum spanning tree analysis based on the sequence-based typing (SBT) of them identified three clonal complexes (CCs); CC1 (105/161, 65.2%), CC2 (22 /161, 13.7%), and CC3 (20/161, 12.4%). CC1 was formed by serogroup (SG) 1 and SG7, whereas CC2 was mainly formed by SG1. All of the CC3 isolates except two strains were SG13. The major sequence types (STs) in CC1 and CC2 were ST1 (88/105, 83.8%) and ST154 (15/22, 68.2%), respectively. These STs are known as typical types of *L. pneumophila* SG1 in Japanese cooling tower. Additionally, we identified 15 strains of ST2603 as the major type in CC3. This ST has not been reported in Japanese cooling tower. Whole genome sequencing (WGS) analysis of the representative strains in the three CCs, which were isolated from various cooling towers over the 10 years, elucidated high clonal population of *L. pneumophila* in Japanese cooling tower. Moreover, it revealed that the strains of CC2 are phylogenetically distant compared to those of CC1 and CC3, and belonged to *L. pneumophila* subsp. *fraseri*.

## 1. Introduction

*Legionella pneumophila* is a major pathogen causing Legionnaires’ disease, whose severity varies from a mild febrile illness to a serious and potentially fatal form of pneumonia [1,2]. *L. pneumophila* is a ubiquitous bacterium generally found in natural freshwater environments, but can also contaminate many artificially made water systems, such as cooling towers, baths, showers, spa pools, decorative fountains, and hot- and cold-potable water systems of large buildings [3]. Legionnaires’ disease is caused by the inhalation of aerosols containing *Legionella*. It is important to monitor the contamination of water systems with these bacteria to prevent legionellosis.

In Japan, several outbreaks of Legionnaires ‘disease associated with public bath facilities have been reported. Public baths are known to be major sources of infection in Japan [4]. However, it is likely that there are many unidentified sources of *Legionella* infections. In addition, cooling towers contaminated with *Legionella* have caused large community outbreaks in several countries [5,6,7,8,9]. Lijie et al. have reported high prevalence and genetic polymorphism in industrial cooling towers in China [10]. Amemura-Maekawa et al. elucidated the differences in the distribution of sequence types (STs) of *L. pneumophila* SG1 isolated from bathwater, cooling tower water, and soil in Japan [11]. However, there exists no surveillance system in cooling towers in Japan focusing on the population structure of *L. pneumophila*.

We have surveyed the presence of Legionella in large buildings and hospital cooling towers in Kobe city, Japan, with yearly regular monitoring. We have collected *Legionella* strains from cooling towers through our routine environmental monitoring from 2003–2012. In this study, we investigated the population structure and genetic characteristics of *L. pneumophila* in cooling towers water system over a period of 10 years, using sequence-based typing (SBT) methods. The detailed characteristics of the representative isolates from the major clonal groups were further identified by whole genome sequencing (WGS).

## 2. Materials and Methods

### 2.1. Bacterial Strains

A total of 161 *L. pneumophila* strains isolated from cooling towers in 62 facilities, including large buildings and hospitals in Kobe City region, Japan from 2003 to 2012, were analyzed in this study (Table S1). Locations and numbers of cooling towers are listed as follows: hospitals (*n* = 36); office buildings (*n* = 27); accommodations (*n* = 21); multipurpose buildings (*n* = 12); apartment houses (*n* = 10); shopping centers (*n* = 8); factories (*n* = 5); schools (*n* = 4); wedding halls (*n* = 2); golf clubs (*n* = 2); public facility (*n* = 1); and unknown (*n* = 33), in which the location of cooling towers could not be identified. We collected *L. pneumophila* strains by using one of two methods. One method was that water samples (500 mL) were filtrated with 0.2 μm pore-size polycarbonate membrane (catalog no.GTTP04700; Millipore, Billerica, MA, USA) and the membrane was resuspended in 5 mL of distilled water. The other was that water samples (200 ml) were centrifuged at 6000 rpm for 30 min at 15 °C and precipitation was resuspended in 2 mL of distilled water. After the concentrated samples were mixed with equal volumes of 0.2 mol/liter KCl-HCl buffer (pH 2.2) for 5 min at room temperature, they were spread onto glycine-vancomycin-polymyxin B cycloheximide agar plates (Oxoid, Basingstoke, Hampshire, United Kingdom) and modified Wadowsky Yee agar plates (Oxoid, Basingstoke, Hampshire, United Kingdom). The agar plates were incubated at 36 °C for 7 days in a moist chamber. Colonies were subcultured onto buffered charcoal yeast extract agar plates (Oxoid, Basingstoke, Hampshire, United Kingdom) and identified as *Legionella* using L-cysteine requirement test and an antiserum kit (Denka Seiken, Japan). The Bacterial isolates from buffered charcoal yeast extract agar plates were suspended in 100 µL TE buffer and incubated for 10 min at 95 °C. After centrifugation to remove cell debris, the supernatant was promptly used as DNA template for the PCRs.

### 2.2. SBT

Genotyping was conducted by the SBT method of the European working group for *Legionella* infections (EWGLI) using 7 genes (*flaA*, *pliE*, *asd*, *mip*, *mompS*, *proA*, and *neuA*), as described previously [12,13,14]. The SBT database that was available on the EWGLI website was used for nucleotide analysis, and the sequences were compared with those in the SBT database, which were also available on the website (http://www.hpa-bioinformatics.org.uk/legionella/legionella_sbt/php/sbt_homepage.php).

### 2.3. Data Analysis

A minimum spanning tree (MST) based on SBT types was constructed using Bionumerics software (Bionumerics ver.7.5; Applied Math., Sint-Martens-Latem, Beigium). An MST had categorical coefficients of similarity and a priority rule for the highest number of single-locus variants as parameters, which were used to indicate differences in the number of loci among operational taxonomic units.

### 2.4. Genome Sequencing of the 25 L. pneumophila Strains

Twenty-five representative strains with different separation locations, years, and STs were selected and subjected to genomic analysis (Table 2). WGS DNA libraries were constructed using a QIAseq FX DNA Library kit (QIAGEN, Hilden, Germany), and paired-end sequences (2 × 300 bp) were generated using the MiSeq system (Illumina, San Diego, CA, USA) with a MiSeq reagent kit v.3 (Illumina). All of the generated reads were assembled into contigs using A5-miseq [15]. For comparative analysis, a core SNP matrix and maximum-likelihood phylogenetic tree were generated using kSNP (v. 3.0) with a k-mer size of 19 [16]. Moreover, the degree of pairwise genome-based relatedness was calculated as an average nucleotide identity (ANI) value following the BLAST-based ANI calculation method using JSpecies software [17]. The genomic sequences of *L. pneumophila* Philadelphia and Paris strains (accession no. AE017354 and CR628336) were obtained from the NCBI database and were used as references. Additionally, two genomic sequences of *L. pneumophila* subsp. *fraseri* strains (accession no. CP017457 and CP017458) were obtained from NCBI database and used as an outgroup.

### 2.5. Nucleotide Sequence Accession Number

The WGS reads are available from the DDBJ/EMBL/GenBank Sequence Read Archive under accession number DRA007772 and DRA008124.

## 3. Results

### 3.1. Distribution of the SGs of L. pneumophila

Among the 161 *L. pneumophila* strains, SG1 was the most frequently isolated SG, accounting for 70.8% (114/161) of the strains, followed by SG13 (18/161, 11.2%), SG7 (13/161, 8.1%), and SG UT (untypable) (7/161, 4.3%) (Table 1).

### 3.2. SBT Analysis of L. pneumophila

**Figure 1:**
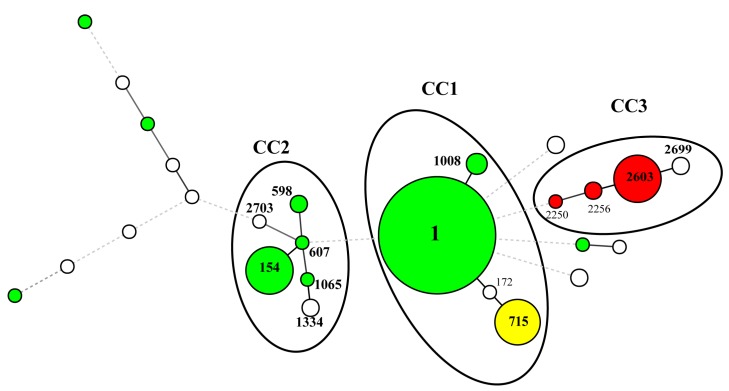
A minimum spanning tree of 161 *L. pnuemohila* strains from cooling towers in Kobe, Japan, based on SBT. The strains belonged to serogroup (SG)1 (*n* = 114), SG13 (*n* = 18), SG7 (*n* = 13) and the other SGs (*n* = 16) were shown as the circles with green, red, yellow, and white respectively. The circle sizes are proportional to the numbers of isolates sharing an identical pattern. The length of the thin line is proportional with single-, double- or triple-locus variants. The dotted lines indicate four loci variants or higher. The clonal complexes (CCs) (CC1, CC2, and CC3) generated with single- and double variants are indicated by the circle surrounding the ST. Whereas KL852 was assigned to ST2703, which differed from ST607 in triple-locus, the strain was included in CC2.

The 161 isolates were differentiated by SBT into 26 different STs (Table 1). The profiles of 20 of the 26 STs could be found in the EWGLI SBT database. Eighty-eight isolates belonged to ST1 (54.7%), which was the most frequently occurring ST, followed by ST2603 (15/161, 9.3%), ST154 (15/161, 9.3%), and ST715 (13/161, 8.1%) (Table 1). Table 1 shows the relationship between SGs and STs. MST-based SBT analysis identified three CCs (Figure 1). Of the 3 CCs, CC1, which was the most prevalent clonal group, consisted of 105 isolates assigned to ST1, ST715, ST1008, and ST172. CC2 was the second-most prevalent clonal group, consisting of 22 isolates that belonged to ST154, ST598, ST607, ST1065, ST1334, and ST2703.

The other CC3 (ST2603, ST2256, ST2250, and ST2699) consisted of 20 isolates. CC1 was formed by SG1 and SG7, while CC2 was mainly formed by SG1. SG13 isolates were contained in CC3. These results suggested that the specific genotypes in CC1, CC2, and CC3 are found in the cooling towers of Japan.

### 3.3. Whole Genome Analysis

**Figure 2:**
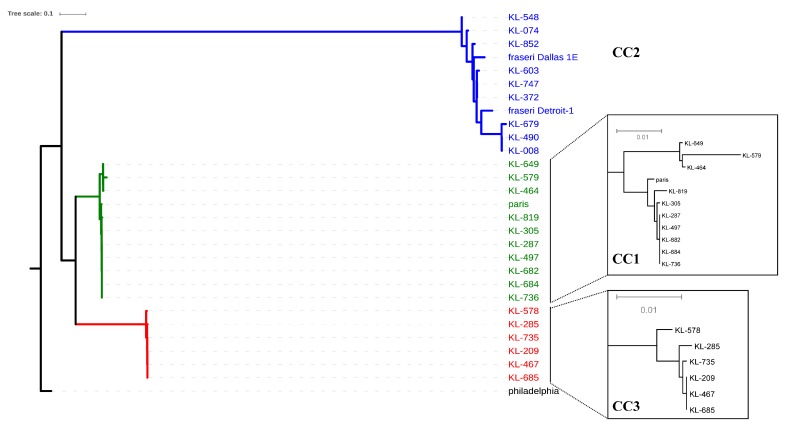
Phylogenetic tree based on a 32586-core-SNP matrixgenome-wide SNPs among the main strains assigned to the three CCs listed in Table 2. A maximum-likelihood tree was constructed using kSNP 3.0 [16]. The lineages including CCs are highlighted by green (CC1), blue (CC2), and red (CC3). Insets show subtrees of CC1 and CC3 isolates.

To clarify the genetic relatedness among the strains belonging to the three CCs isolated from the different cooling towers in Japan during separate years, the whole-genome sequences were compared. In the phylogenetic tree based on the core genome SNPs, the three CCs were divided into three clades (Figure 2). Despite of KL852 assigned to ST2703, which differed from ST607 in triple-locus, the core genome SNPs phylogenetic tree indicated that KL852 was included in CC2 cluster (Table 2 and Figure 2).

For each clades containing each CCs (CC1, CC2, and CC3), 1093, 4558, and 245 of core SNPs were detected, respectively. CC1 contained the *L. pneumophila* strain Paris assigned to ST1. The CC1 cluster strains were classified into two sub-clades. In the sub-clades, closely related strain groups (e.g., KL464 and KL649; KL287, KL305, KL497, KL682, KL684, and KL736), with the number of SNPs being 0 or 1, were detected despite the separation years and/or sites. Likewise, the four strains of CC3 (KL209, KL467, KL685, and KL735) isolated from spatiotemporally separated cooling towers were also closely related to each other with 0 or 1 core SNPs. Surprisingly, even strains isolated from the same cooling towers were classified as CC1 or CC3, suggesting that several specific genetic strains of *L. pneumophila* could coexist in the same environment.

The phylogenetic tree based on the core genome SNPs demonstrated that CC2 is phylogenetically distant compared to CC1 and CC3 (Figure 2). Therefore, we calculated the ANI values among the strains. The ANI values among reference strains Paris and Philadelphia and our strains belonging to CC1 and CC3 ranged between 96.0 and 99.9%. These ANI values were higher than the 95% cut-off ANI value for bacterial species proposed by Goris et al. [18]. On the other hand, the ANI values in CC1 versus CC2 and CC3 versus CC2 were well below (91.8–92.8%) the proposed cut-off ANI value (95%) for bacterial species. Furthermore, the ANI value between CC2 and *L. pneumophila subsp. fraseri* was in the range of 98.4–99.8%. Therefore, when combined with the same cluster in the phylogenetic tree, the isolates of CC2 are concluded to belong to *L. pneumophila* subsp. *fraseri.*

## 4. Discussion

In this study, we analyzed *L. pneumophila* isolates obtained from cooling towers in Kobe city, Japan, during the past 10 years, by SBT and WGS. We revealed that the three high clonal groups, CC1, CC2, and CC3, have colonized the cooling towers in Japan for many years. Furthermore, we found that CC1 and CC3 strains could coexist in the same environment: e.g., KL287 (CC1) and KL285 (CC3); KL464 (CC1) and KL467 (CC3); KL579 (CC1) and KL578 (CC3); KL684 (CC1) and KL685 (CC3); KL736 (CC1) and KL735 (CC3) (Table 2). Our finding was suggested that these limited genetic groups of *L. pneumophila* might have been continuously inhabitant in Japanese cooling towers. Lijie et al., have reported high genetic polymorphism in industrial cooling towers in China [18]. However, we found lower genetic diversity in cooling towers in Japan through our monitoring over many years.

The major SGs constituting CC1, CC2, and CC3 were SG1, SG13, and SG7. In Japan, the main infection sources of legionellosis are hot springs and public baths [4]. SG1 has been found to be the major SG involved in bathwater-associated cases of legionellosis in Japan [19]. SG1 is also the predominant SG causing clinical Legionnaires’ disease cases, while legionellosis caused by SG13 and SG7 is very rare in Japan [20]. However, an outbreak of Legionnaires’ disease caused by SG13 and SG1 *L. pneumophila* strains in a spa house was recently reported in Japan [21]. Previous studies have revealed the variation of SGs and STs in bath water and shower water in public bath facilities and have reported them as infection sources in legionellosis cases [22]. On the other hand, as per a previous study and our findings, some definite SGs, SG1, SG13, and SG7, occupy niches in the cooling tower [23]. Therefore, it could be suggested that the distribution of SGs differed between public baths and cooling towers in Japan. In addition, a previous study has reported that the *flaA* genotypes in cooling towers were lower diversity than in bath water, and that *flaA*1 or *flaA*11 were the predominant *flaA* genotypes in cooling towers in Japan [23]. Similarly, in our study, we found that the dominant CC1 and CC2 strains harbored *flaA*1 and *flaA*11, respectively (Table S1). Conclusively, it can be suggested that limited genotypes colonize the cooling towers in Japan. This limited diversity might be due to the sources and properties of cooling towers water, which is chemically more homogeneous than public baths water from hot springs with different composition [23].

In a previous study, the distribution of STs in *L. pneumophila* SG1 differed between the water sources: hot springs, cooling towers, potable water systems, and soils in Japan, China, and South Korea [10,24,25]. Among the 26 STs detected in the 161 isolates from the cooling tower, ST1 in CC1 and ST154 in CC2 have been previously identified as STs in *L. pneumophila* SG1 isolated from a cooling tower in Japan [10]. ST1 (88/161, 54.7%) was the most dominant ST in this study. ST1 has been known to be the most prevalent worldwide [26,27,28]. Recent research has shown that ST1 is the major ST in clinical *L. pneumophila* in Japan [20]. The SG1-specific gene *lag−1* is known to be a pathogenic marker [29]. Only two strains, ST2 and ST40, among the 114 SG1 strains were *lag−1* positive, whereas the most prevalent ST1 isolated in this study were *lag−1* negative (Table S1). Since the ST1 strains lacking *lag−1* were reported as major clinical ST previously, it is considered necessary to focus on ST1 as sequence type which is at risk for clinical infection [20].

Phylogenetic trees based on the core genome SNPs showed that CC2 separated from CC1 and CC3. All 22 isolates belonging to the CC2 group possessed *pilE*14, consistent with a previous report [20]. Based on a genome phylogenetic tree, we showed that the CC2 strains are present in the same cluster as *L. pneumophila* subsp. *fraseri* strains. According to a previous study, the SBT pattern in *L. pneumophila* subsp. *fraseri* strains has been identified as the consensus pattern **11**-x-**16**-x-x-**13**-x (***flaA**-pliE-**asd**-mip-mompS-**proA**-neuA*) [30]. ST154, ST598, ST607, ST1065, ST1334, and ST2703 included in CC2 also exhibited the **11**-x-**16**-x-x-**13**-x pattern. Using a combination of phylogeny and genome sequence comparison, CC2 has been identified to belong to *L. pneumophila* subsp. *fraseri*.

In this study, core SNPs were identified using kSNP software to further resolve the genome sequences. WGS based genotypes correlated with the ST classification by SBT. Hundreds of core SNPs are detected between strains of different genetic lineage. Furthermore, core SNPs genotypes were elucidated the genomic proximity and stability between strains within each CC, despite the difference in separation years and separation sites. Brian et al. have reported that isolates associated with specific *Legionella* outbreaks differed by <5 core SNPs and formed outbreak-specific clades [31]. In our research, despite the differences in separation years and separation sites, there are strains in which only 0 or 1 core SNP are detected among the strains within each CC (such as, KL287, KL305, KL497, KL682, KL684, and KL736). Our results are suggested that the identical genotype in three genetic lineages are adapted to cooling towers in Japan.

## 5. Conclusions

In conclusion, we found three high clonal groups, CC1, CC2, and CC3, which had colonized cooling towers in Japan over our ten year monitoring period. We elucidated that CC2 is phylogenetically distant compared to CC1 and CC3, and belonged to *L. pneumophila* subsp. *fraseri.* Our findings could be helpful for estimating the sources of infection in Japan and developing prevention strategies.

## Figures and Tables

**Table 1:** Distribution of SGs and STs of 161 *L. pneumophila* isolated in cooling tower, Kobe, Japan form 2003–2012.

SG	No. of Isolates (%)		STs (No. of Isolates)
SG1	114	(70.8)	ST1 (88), ST154 (15), ST1008 (3), ST598 (2), ST2 (1), ST40 (1), ST45 (1), ST59 (1), ST607 (1), ST1065 (1)
SG13	18	(11.2)	ST2603 (15), ST2256 (2), ST2250 (1)
SG7	13	(8.1)	ST715 (13)
SG UT	7	(4.3)	ST1334 (2), ST2699 * (2), ST172 (1), ST1916 (1), ST2701 * (1)
SG5	4	(2.5)	ST2700 * (2), ST2704 * (2)
SG6	2	(1.2)	ST68 (1), ST242 (1)
SG2	1	(0.6)	ST2702 * (1)
SG8	1	(0.6)	ST1324 (1)
SG9	1	(0.6)	ST2703 * (1)

* We identified as new sequence types (STs) in sequence-based typing (SBT) database.

**Table 2:** Information of the 25 strains assigned to three complexes on genome analysis.

Complexes	No. of Strain	Years	Site in CT ^a^	SG	ST	DDBJ Accession No. of Read Data
CC1	KL287	2006	F	1	1	DRR163670
	KL305	2006	A	1	1	DRR163671
	KL464	2008	A	1	1	DRR163673
	KL497	2008	E	1	1	DRR163675
	KL579	2009	H	1	1	DRR163677
	KL649	2010	A	1	1	DRR163679
	KL682	2010	M	1	1008	DRR170776
	KL684	2010	D	1	1	DRR163680
	KL736	2011	J	1	1	DRR163683
	KL819	2012	Q	7	715	DRR170777
CC2	KL008	2003	K	UT	1334	DRR170771
	KL074	2004	L	1	607	DRR170772
	KL372	2007	C	1	154	DRR163672
	KL490	2008	N	UT	1334	DRR170773
	KL548	2009	O	1	598	DRR170774
	KL603	2009	I	1	154	DRR163678
	KL679	2010	P	1	1065	DRR170775
	KL747	2011	G	1	154	DRR163684
	KL852 ^b^	2012	L	9	2703	DRR170778
CC3	KL209	2005	B	13	2603	DRR163668
	KL285	2006	F	UT	2699	DRR163669
	KL467	2008	A	13	2603	DRR163674
	KL578	2009	H	13	2603	DRR163676
	KL685	2010	D	13	2603	DRR163681
	KL735	2011	J	13	2603	DRR163682

^a^ Locations of cooling towers are as follows: hospital (A); unknown (B, K); office buildings (C, F, L, Q); apartment house (D); accommodations (E, H, J, M, N, O); shopping center (G); wedding hall (I). ^b^ Whereas KL852 was assigned to ST2703, which differed from ST607 in triple-locus, the strain was included in CC2.

## Supplementary Materials

The following are available online at https://www.mdpi.com/1660-4601/16/9/1664/s1, Table S1: Information of the 161 strains in this study.

## Abbreviations

CC	Clonal complex
SBT	Sequence-based typing
WGS	Whole genome sequencing
UT	Untypable
ST	Sequence type
SG	Serogroup
ANI	Average nucleotide identity
SNP	Single nucleotide polymorphism
