# Supplementary material for: Analysis of Genetic Characterization and Clonality of Legionella pneumophila Isolated from Cooling Towers in Japan
Source: Int J Environ Res Public Health. 2019 May 13;16(9):1664. doi: 10.3390/ijerph16091664 (PMC6540132; doi:10.3390/ijerph16091664)
Supplement: Supplementary file 1 [file ijerph-16-01664-s001.pdf]

Table S1. Information of the 161 strains in this study.

No. of Strain	Years	Sites	Serogroup (SG)	SBT Profile							ST	*lag-1*	Clonal Complexes (CCs)
				flaA	*pliE*	*asd*	*mip*	*mompS*	*proA*	*neuA*			
KL5	2003	unknown	1	1	4	3	1	1	1	1	1	negative	CC1
KL8	2003	unknown	UT	11	14	16	25	7	13	206	1334	negative	CC2
KL11	2003	unknown	13	16	21	33	37	31	1	222	2603	negative	CC3
KL12	2003	unknown	1	1	4	3	1	1	1	1	1	negative	CC1
KL15	2003	unknown	1	1	4	3	1	1	1	1	1	negative	CC1
KL16	2003	unknown	13	16	21	33	37	31	1	222	2603	negative	CC3
KL17	2003	unknown	1	11	14	16	16	15	13	2	154	negative	CC2
KL18	2003	unknown	13	16	21	33	37	31	1	222	2603	negative	CC3
KL22	2003	hospital	1	1	4	3	1	1	1	1	1	negative	CC1
KL23	2003	hospital	1	1	4	3	1	1	1	1	1	negative	CC1
KL25	2003	hospital	1	1	4	3	1	1	1	1	1	negative	CC1
KL30	2003	hospital	1	1	4	3	1	1	1	1	1	negative	CC1
KL32	2003	hospital	1	1	4	3	1	1	1	1	1	negative	CC1
KL74	2004	office building	1	11	14	16	16	15	13	1	607	negative	CC2
KL80	2004	hospital	1	1	4	3	1	1	1	1	1	negative	CC1
KL82	2004	hospital	1	1	4	3	1	1	1	1	1	negative	CC1
KL86	2004	hospital	1	1	4	3	1	1	1	1	1	negative	CC1
KL89	2004	office building	1	11	14	16	16	15	13	2	154	negative	CC2
KL91	2004	office building	1	11	14	16	16	15	13	2	154	negative	CC2
KL99	2004	unknown	1	1	4	3	1	1	1	1	1	negative	CC1
KL101	2004	unknown	1	1	4	3	1	1	1	1	1	negative	CC1
KL105	2004	unknown	1	1	4	3	1	1	1	1	1	negative	CC1
KL117	2004	hospital	1	1	4	3	1	1	1	1	1	negative	CC1
KL118	2004	unknown	1	1	4	3	1	1	1	1	1	negative	CC1
KL120	2004	unknown	1	1	4	3	1	1	1	1	1	negative	CC1
KL124	2004	unknown	1	11	14	16	16	15	13	2	154	negative	CC2
KL125	2004	hospital	1	1	4	3	1	1	1	1	1	negative	CC1
KL127	2004	hospital	1	1	4	3	1	1	1	1	1	negative	CC1
KL132	2004	unknown	1	1	4	3	1	1	1	1	1	negative	CC1
KL133	2004	office building	5	8	49	34	9	12	8	209	2700	negative	
KL140	2004	unknown	1	1	4	3	1	1	1	1	1	negative	CC1

KL142	2004	unknown	7	1	4	3	19	1	1	2	715	negative	CC1
KL152	2004	unknown	1	1	4	3	1	1	1	1	1	negative	CC1
KL153	2004	unknown	7	1	4	3	19	1	1	2	715	negative	CC1
KL154	2004	unknown	1	1	4	3	1	1	1	1	1	negative	CC1
KL161	2005	unknown	7	1	4	3	19	1	1	2	715	negative	CC1
KL162	2005	unknown	7	1	4	3	19	1	1	2	715	negative	CC1
KL198	2005	unknown	7	1	4	3	19	1	1	2	715	negative	CC1
KL200	2005	unknown	7	1	4	3	19	1	1	2	715	negative	CC1
KL208	2005	unknown	1	1	4	3	1	1	1	1	1	negative	CC1
KL209	2005	unknown	13	16	21	33	37	31	1	222	2603	negative	CC3
KL210	2005	unknown	1	1	4	3	1	1	1	1	1	negative	CC1
KL211	2005	unknown	6	3	13	1	28	14	9	3	68	negative	
KL212	2005	unknown	7	1	4	3	19	1	1	2	715	negative	CC1
KL225	2005	unknown	1	1	4	3	1	1	1	1	1	negative	CC1
KL234	2005	unknown	1	1	4	3	1	1	1	1	1	negative	CC1
KL235	2005	unknown	1	1	4	3	1	1	1	1	1	negative	CC1
KL237	2005	unknown	1	1	4	3	1	1	1	1	1	negative	CC1
KL276	2006	shopping center	1	11	14	16	16	15	13	2	154	negative	CC2
KL278	2006	office building	13	16	21	33	37	31	1	222	2603	negative	CC3
KL284	2006	office building	1	1	4	3	1	1	1	1	1	negative	CC1
KL285	2006	office building	UT	16	21	33	37	31	1	215	2699	negative	CC3
KL287	2006	office building	1	1	4	3	1	1	1	1	1	negative	CC1
KL301	2006	shopping center	7	1	4	3	19	1	1	2	715	negative	CC1
KL302	2006	shopping center	7	1	4	3	19	1	1	2	715	negative	CC1
KL304	2006	shopping center	7	1	4	3	19	1	1	2	715	negative	CC1
KL305	2006	hospital	1	1	4	3	1	1	1	1	1	negative	CC1
KL306	2006	hospital	1	1	4	3	1	1	1	1	1	negative	CC1
KL307	2006	hospital	1	1	4	3	1	1	1	1	1	negative	CC1
KL308	2006	hospital	1	1	4	3	1	1	1	1	1	negative	CC1
KL309	2006	hospital	1	1	4	3	1	1	1	1	1	negative	CC1
KL310	2006	hospital	1	1	4	3	1	1	1	1	1	negative	CC1
KL311	2006	hospital	1	1	4	3	1	1	1	1	1	negative	CC1
KL312	2006	hospital	1	1	4	3	1	1	1	1	1	negative	CC1
KL348	2007	multipurpose buildings	1	1	4	3	1	1	1	1	1	negative	CC1
KL354	2007	hospital	1	1	4	3	1	1	1	1	1	negative	CC1
KL355	2007	hospital	1	1	4	3	1	1	1	1	1	negative	CC1

KL356	2007	hospital	13	16	21	33	37	31	1	222	2603	negative	CC3
KL358	2007	hospital	13	16	21	33	37	31	1	222	2603	negative	CC3
KL361	2007	accommodation	1	1	4	3	1	1	1	1	1	negative	CC1
KL362	2007	accommodation	13	28	21	33	37	41	1	1	2250	negative	CC3
KL372	2007	office building	1	11	14	16	16	15	13	2	154	negative	CC2
KL373	2007	office building	1	11	14	16	16	15	13	2	154	negative	CC2
KL374	2007	multipurpose buildings	1	1	4	3	1	1	1	1	1	negative	CC1
KL381	2007	multipurpose buildings	1	11	14	16	16	15	13	2	154	negative	CC2
KL382	2007	office building	1	11	14	16	16	15	13	2	154	negative	CC2
KL383	2007	office building	1	11	14	16	16	15	13	2	154	negative	CC2
KL384	2007	office building	1	11	14	16	16	15	13	2	154	negative	CC2
KL385	2007	factory	5	12	23	13	54	32	45	15	2704	negative	
KL400	2007	factory	UT	6	10	15	28	17	14	207	1916	negative	
KL410	2007	factory	5	12	23	13	54	32	45	15	2704	negative	
KL412	2007	factory	1	6	10	19	3	19	4	9	2	positive	
KL413	2007	factory	UT	6	10	3	78	9	4	218	2701	negative	
KL463	2008	hospital	1	1	4	3	1	1	1	1	1	negative	CC1
KL464	2008	hospital	1	1	4	3	1	1	1	1	1	negative	CC1
KL465	2008	hospital	13	16	21	33	37	31	1	222	2603	negative	CC3
KL467	2008	hospital	13	16	21	33	37	31	1	222	2603	negative	CC3
KL489	2008	accommodation	1	1	4	3	1	1	1	1	1	negative	CC1
KL490	2008	accommodation	UT	11	14	16	25	7	13	206	1334	negative	CC2
KL491	2008	accommodation	1	1	4	3	1	1	1	1	1	negative	CC1
KL492	2008	accommodation	1	1	4	3	1	1	1	1	1	negative	CC1
KL493	2008	accommodation	13	16	21	33	37	41	1	222	2256	negative	CC3
KL494	2008	accommodation	UT	1	4	3	1	1	1	2	172	negative	CC1
KL496	2008	accommodation	1	1	4	3	1	1	1	1	1	negative	CC1
KL497	2008	accommodation	1	1	4	3	1	1	1	1	1	negative	CC1
KL498	2008	accommodation	1	1	4	3	1	1	1	1	1	negative	CC1
KL548	2009	accommodation	1	11	14	16	10	15	13	11	598	negative	CC2
KL550	2009	accommodation	UT	16	21	33	37	31	1	215	2699	negative	CC3
KL551	2009	multipurpose buildings	1	1	4	3	1	1	1	1	1	negative	CC1
KL566	2009	hospital	1	1	4	3	1	1	1	1	1	negative	CC1
KL567	2009	hospital	1	1	4	3	1	1	1	1	1	negative	CC1
KL568	2009	hospital	1	1	4	3	1	1	1	1	1	negative	CC1
KL570	2009	hospital	13	16	21	33	37	31	1	222	2603	negative	CC3

KL571	2009	hospital	1	1	4	3	1	1	1	1	1	negative	CC1
KL572	2009	hospital	1	1	4	3	1	1	1	1	1	negative	CC1
KL578	2009	accommodation	13	16	21	33	37	31	1	222	2603	negative	CC3
KL579	2009	accommodation	1	1	4	3	1	1	1	1	1	negative	CC1
KL580	2009	multipurpose buildings	1	1	4	3	1	1	1	1	1	negative	CC1
KL582	2009	school	1	1	4	3	1	1	1	1	1	negative	CC1
KL583	2009	school	13	16	21	33	37	41	1	222	2256	negative	CC3
KL587	2009	multipurpose buildings	1	1	4	3	1	1	1	1	1	negative	CC1
KL589	2009	multipurpose buildings	1	1	4	3	1	1	1	1	1	negative	CC1
KL598	2009	multipurpose buildings	1	1	4	3	1	1	1	1	1	negative	CC1
KL602	2009	multipurpose buildings	1	1	4	3	1	1	1	1	1	negative	CC1
KL603	2009	wedding hall	1	11	14	16	16	15	13	2	154	negative	CC2
KL604	2009	wedding hall	1	1	4	3	1	1	1	1	1	negative	CC1
KL612	2009	multipurpose buildings	1	1	4	3	1	1	1	1	1	negative	CC1
KL614	2009	multipurpose buildings	1	1	4	3	1	1	1	1	1	negative	CC1
KL616	2009	multipurpose buildings	1	1	4	3	1	1	1	1	1	negative	CC1
KL649	2010	hospital	1	1	4	3	1	1	1	1	1	negative	CC1
KL650	2010	hospital	1	1	4	3	1	1	1	1	1	negative	CC1
KL652	2010	hospital	1	1	4	3	1	1	1	1	1	negative	CC1
KL659	2010	apartment house	1	1	4	3	1	1	1	1	1	negative	CC1
KL660	2010	apartment house	6	3	10	1	28	1	9	3	242	negative	
KL661	2010	apartment house	1	1	4	3	1	1	1	1	1	negative	CC1
KL662	2010	accommodation	1	1	4	3	1	1	41	1	1008	negative	CC1
KL663	2010	office building	1	11	14	16	10	15	13	11	598	negative	CC2
KL672	2010	apartment house	1	1	4	3	1	1	1	1	1	negative	CC1
KL673	2010	apartment house	13	16	21	33	37	31	1	222	2603	negative	CC3
KL674	2010	apartment house	1	1	4	3	1	1	41	1	1008	negative	CC1
KL678	2010	school	1	11	14	16	16	15	13	2	154	negative	CC2
KL679	2010	accommodation	1	11	14	16	25	7	13	1	1065	negative	CC2
KL680	2010	accommodation	1	1	4	3	1	1	1	1	1	negative	CC1
KL682	2010	accommodation	1	1	4	3	1	1	41	1	1008	negative	CC1
KL683	2010	apartment house	1	1	4	3	1	1	1	1	1	negative	CC1
KL684	2010	apartment house	1	1	4	3	1	1	1	1	1	negative	CC1
KL685	2010	apartment house	13	16	21	33	37	31	1	222	2603	negative	CC3
KL686	2010	shopping center	13	16	21	33	37	31	1	222	2603	negative	CC3
KL687	2010	office building	1	1	4	3	1	1	1	1	1	negative	CC1

KL689	2010	office building	7	1	4	3	19	1	1	2	715	negative	CC1
KL690	2010	office building	2	3	6	1	7	14	11	6	2702	negative	
KL701	2010	office building	1	1	4	3	1	1	1	1	1	negative	CC1
KL702	2010	school	1	11	14	16	16	15	13	2	154	negative	CC2
KL703	2010	apartment house	1	1	4	3	1	1	1	1	1	negative	CC1
KL734	2011	golf club	1	1	4	3	1	1	1	1	1	negative	CC1
KL735	2011	accommodation	13	16	21	33	37	31	1	222	2603	negative	CC3
KL736	2011	accommodation	1	1	4	3	1	1	1	1	1	negative	CC1
KL737	2011	golf club	1	1	4	3	1	1	1	1	1	negative	CC1
KL747	2011	shopping center	1	11	14	16	16	15	13	2	154	negative	CC2
KL748	2011	shopping center	1	3	6	1	14	14	9	11	40	positive	
KL749	2011	shopping center	1	5	1	22	26	6	10	12	45	negative	
KL750	2011	office building	5	8	49	34	9	12	8	209	2700	negative	
KL797	2012	office building	1	7	6	17	3	13	11	11	59	negative	
KL818	2012	office building	1	1	4	3	1	1	1	1	1	negative	CC1
KL819	2012	office building	7	1	4	3	19	1	1	2	715	negative	CC1
KL850	2012	public facility	8	5	1	22	30	6	10	203	1324	negative	
KL851	2012	office building	1	1	4	3	1	1	1	1	1	negative	CC1
KL852	2012	office building	9	3	14	16	28	15	13	6	2703	negative	CC2
KL853	2012	office building	1	1	4	3	1	1	1	1	1	negative	CC1
KL854	2012	office building	7	1	4	3	19	1	1	2	715	negative	CC1
KL855	2012	office building	1	1	4	3	1	1	1	1	1	negative	CC1

* The strains used in genome analysis are highlighted by gray.
